# Single-Molecule Studies of the Im7 Folding Landscape

**DOI:** 10.1016/j.jmb.2010.02.048

**Published:** 2010-04-23

**Authors:** Sara D. Pugh, Christopher Gell, D. Alastair Smith, Sheena E. Radford, David J. Brockwell

**Affiliations:** Astbury Centre for Structural Molecular Biology, Institute of Molecular and Cellular Biology, University of Leeds, Leeds LS2 9JT, UK

**Keywords:** Im, immunity protein, MD, molecular dynamics, smFRET, single-molecule fluorescence energy transfer, Im7, folding, intermediate, fluorescence resonance energy transfer, unfolded ensemble

## Abstract

Under appropriate conditions, the four-helical Im7 (immunity protein 7) folds from an ensemble of unfolded conformers to a highly compact native state via an on-pathway intermediate. Here, we investigate the unfolded, intermediate, and native states populated during folding using diffusion single-pair fluorescence resonance energy transfer by measuring the efficiency of energy transfer (or proximity or *P* ratio) between pairs of fluorophores introduced into the side chains of cysteine residues placed in the center of helices 1 and 4, 1 and 3, or 2 and 4. We show that while the native states of each variant give rise to a single narrow distribution with high *P* values, the distributions of the intermediates trapped at equilibrium (denoted I^eqm^) are fitted by two Gaussian distributions. Modulation of the folding conditions from those that stabilize the intermediate to those that destabilize the intermediate enabled the distribution of lower *P* value to be assigned to the population of the unfolded ensemble in equilibrium with the intermediate state. The reduced stability of the I^eqm^ variants allowed analysis of the effect of denaturant concentration on the compaction and breadth of the unfolded state ensemble to be quantified from 0 to 6 M urea. Significant compaction is observed as the concentration of urea is decreased in both the presence and absence of sodium sulfate, as previously reported for a variety of proteins. In the presence of Na_2_SO_4_ in 0 M urea, the *P* value of the unfolded state ensemble approaches that of the native state. Concurrent with compaction, the ensemble displays increased peak width of *P* values, possibly reflecting a reduction in the rate of conformational exchange among iso-energetic unfolded, but compact conformations. The results provide new insights into the initial stages of folding of Im7 and suggest that the unfolded state is highly conformationally constrained at the outset of folding.

## Introduction

**Fig. 1:**
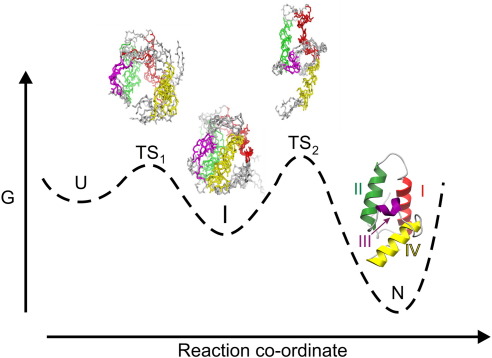
Schematic representation of the folding landscape of Im7 involving a populated on-pathway intermediate. Species on the folding pathway are indicated by the following: U, unfolded state; I, intermediate state; N, native state; TS_1_ and TS_2_, early and rate-determining transition states. Representative members of the ensembles calculated by MD simulations^10,14^ are shown for TS_1_, I, and TS_2_. The structure of native Im7 (Protein Data Bank code 1AYI)^13^ is shown in the native well. The segments forming the four helices in the native state are colored red (helix 1), green (helix 2), purple (helix 3), and yellow (helix 4).

In order to fully understand the folding mechanism of a protein, it is necessary to characterize every species populated along the folding pathway from the initial denatured state to the final native conformation. For several small proteins that fold with a two-state transition, direct characterization of folding is limited to the folded and unfolded states. An increasing number of small single-domain proteins, however, have been shown to fold with multi-state kinetics^1–9^ indicative of the population of partially folded intermediates along the pathway. These proteins provide an opportunity to dissect the structures populated en route to the native state, and both the structural and the dynamical characterization of these species have provided key insights into the organization of structure during protein folding trajectories.^10–12^ Studies of the folding mechanisms of the bacterial DNase-specific immunity proteins, Im7 and Im9, are particularly powerful as, despite their similarity in sequence,^13^ Im9 folds with an apparent two-state transition, while Im7 folds with a three-state transition via an on-pathway populated intermediate (Fig. 1).^5,10,14–17^ φ value analysis and native-state hydrogen exchange experiments have shown that the Im7 intermediate contains three of the four native helices (helices 1, 2, and 4) packed around a specific hydrophobic core that lacks helix 3.^16,18^ Selective destabilization of the native state by mutation (L53A I54A) to such an extent that the intermediate becomes the most stable species at equilibrium^19^ has allowed structural analysis of this partially folded state by NMR.^20^ The intermediate was found to contain native-like secondary structure in helices 1 and 4, partial formation of helix 2, the absence of helix 3, and a fluid, rather than a uniquely structured core.^20^ By using chemical shift analysis, hydrogen exchange, and φ values as restraints for molecular dynamics (MD) simulations, models of the kinetic intermediate have been proposed,^10,14,20^ in which helices 1, 2, and 4 are aligned in a native-like topology but their docking is reorganized to allow nonnative interactions to form between helices 2 and 4, while residues that ultimately form helix 3 remain highly disordered.^10,14^

The extent of conformational heterogeneity within the Im7 folding intermediate is difficult to verify using ensemble techniques since such measurements typically yield parameters that are averaged over the entire ensemble of conformations within each population. By contrast, single-molecule experiments are ideal for the detection and characterization of rarely populated conformations in heterogeneous ensembles.^21–29^ Diffusion single-molecule fluorescence energy transfer (smFRET), in particular, is a powerful technique for studying the structural and dynamic properties of unfolded and folded protein subpopulations at equilibrium.^21,22,27^ Importantly, this technique can be used to quantify and characterize each species within an ensemble, even when populated to just a few percent. We have previously used smFRET to measure the effects of denaturant on the conformational properties of the native and unfolded states of Im9 and demonstrated that the unfolded ensemble becomes significantly compacted at lower denaturant concentrations while the native state shows only minor effects as chaotrope is titrated.^30^

Here, we describe new experiments using diffusion smFRET, designed to directly probe the structural properties of Im7 in its native and trapped intermediate ensembles. We achieve this by measuring the FRET efficiency between dye donor–acceptor pairs introduced at defined points in the protein sequence, chosen to allow us to monitor the relative conformational arrangement and dynamics of each of the four helices at different points in the folding landscape. As well as examining the folded and intermediate states, the low stability of the double-dye-labeled trapped intermediate provides a means of directly monitoring the structural properties of the unfolded state under physiological conditions. Upon addition of kosmotrope under these conditions, the unfolded ensemble is highly compact and displays an increase in the peak width of *P* values, possibly reflecting a reduction in the rate of conformational exchange among iso-energetic unfolded but compact conformations, suggesting that the search process for folding is highly constrained from the onset.

## Results

### Experimental design and characterization of cysteine variants of Im7

In order to investigate the structural properties of the unfolded, intermediate, and folded states of Im7 using smFRET, we identified solvent-exposed residues close to the center of each helix (Q17, V36, Y56, and K70 in helices 1, 2, 3, and 4, respectively), and cysteine residues were then introduced (see Methods) at pairs of these sites in both wild-type Im7 and the trapped intermediate (Im7 L53A I54A, referred to as I^eqm^).^19^ The variants are named according to the helices to which the dyes are attached; for example, Im7 Q17C K70C is referred to as Im7 H1H4. In total, three pairs of variants were studied for Im7 and Im7 I^eqm^ with dye-attachment sites in H1H4, H2H4, and H1H3. To amplify the sensitivity of smFRET to conformational changes in Im7 H1H3, we inserted 15 glycine residues into the loop connecting helix 1 and helix 2, in both Im7 and Im7 I^eqm^. The H1H3 variants are therefore named Im7 GlyH1H3 and Im7 I^eqm^ GlyH1H3. The expansion of this loop has previously been shown to have no effect on the folding mechanism of native Im7 or its folding intermediate (G. Spence and S.E.R., unpublished data).

**Fig. 2:**
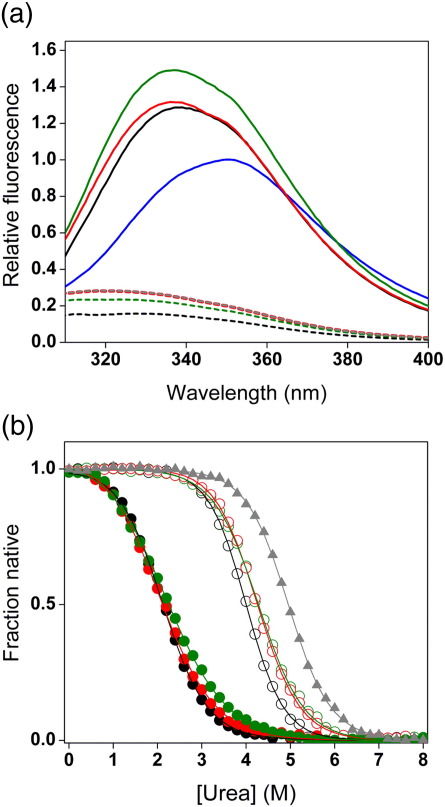
(a) Normalized fluorescence emission spectra of the folded and denatured states of the wild-type Im7 double-cysteine variants and Im7 I^eqm^ double-cysteine variants. The broken black, green, red, and gray lines correspond to Im7 GlyH1H3, Im7 H2H4, Im7 H1H4, and wild-type Im7 in 0 M urea, respectively. The continuous black, green, and red lines correspond to Im7 I^eqm^GlyH1H3, Im7 I^eqm^H2H4, and Im7 I^eqm^H1H4 in 0 M urea, respectively. The continuous blue lines correspond to the average of all the unfolded states in 8 M urea. All signals were normalized to the maximum signal of the denatured state in 8 M urea. (b) Urea-induced equilibrium unfolding curves of the wild-type Im7 double-cysteine variants and I^eqm^ Im7 double-cysteine variants. Denaturation was monitored by tryptophan fluorescence and normalized to the fraction of folded molecules present. Filled circles correspond to I^eqm^ double-cysteine variants, open circles correspond to the wild-type Im7 double-cysteine variants, and filled gray triangles correspond to wild-type Im7. Color coding of the different helical pairs is identical with that shown in (a). The continuous lines show the fits to a two-state transition.

Before labeling with fluorescent dyes, each Im7 variant was characterized using tryptophan fluorescence emission, equilibrium denaturation (Fig. 2; Table 1), and 1D ^1^H NMR (data not shown) to ensure that the mutations introduced had not perturbed the structural properties of the native and intermediate ensembles. The incorporation of cysteine was found not to substantially alter the structure or stability of native or partially unfolded Im7 variants. Thus, the native and intermediate states of all variants were destabilized by ≤ 4.5 kJ mol^− 1^ compared with their wild-type counterparts (Table 1) and all native variants gave rise to tryptophan fluorescence emission spectra that are highly quenched (Fig. 2, broken lines), indicative of the native-like stacking of the sequentially distant His47 and Trp75 pair.^5,15^ By contrast, the fluorescence emission spectrum of all of the trapped intermediate variants were more intense than those of either the unfolded or native states, consistent with formation of the previously identified hyper-fluorescent intermediate state.^5,15^ Finally, 1D ^1^H NMR spectra were similar to those observed previously for each state, confirming that introduction of two cysteine residues did not significantly alter the structure of the intermediate or native states.

Donor and acceptor fluorophores (Alexa 488 and Alexa 594) for FRET were then introduced into each variant by a two-step procedure (see Methods) that yielded proteins labeled with a single donor–acceptor pair. Steady-state anisotropy measurements exciting each fluorophore gave anisotropy values for all variants (both in folded and unfolded states) from 0.10 to 0.19 (Table 1), suggesting that both dyes have a high degree of flexibility in all conditions and, hence, are useful as FRET probes.^27,31^

### Conformational ensembles of the native and intermediate states of Im7

**Fig. 3:**
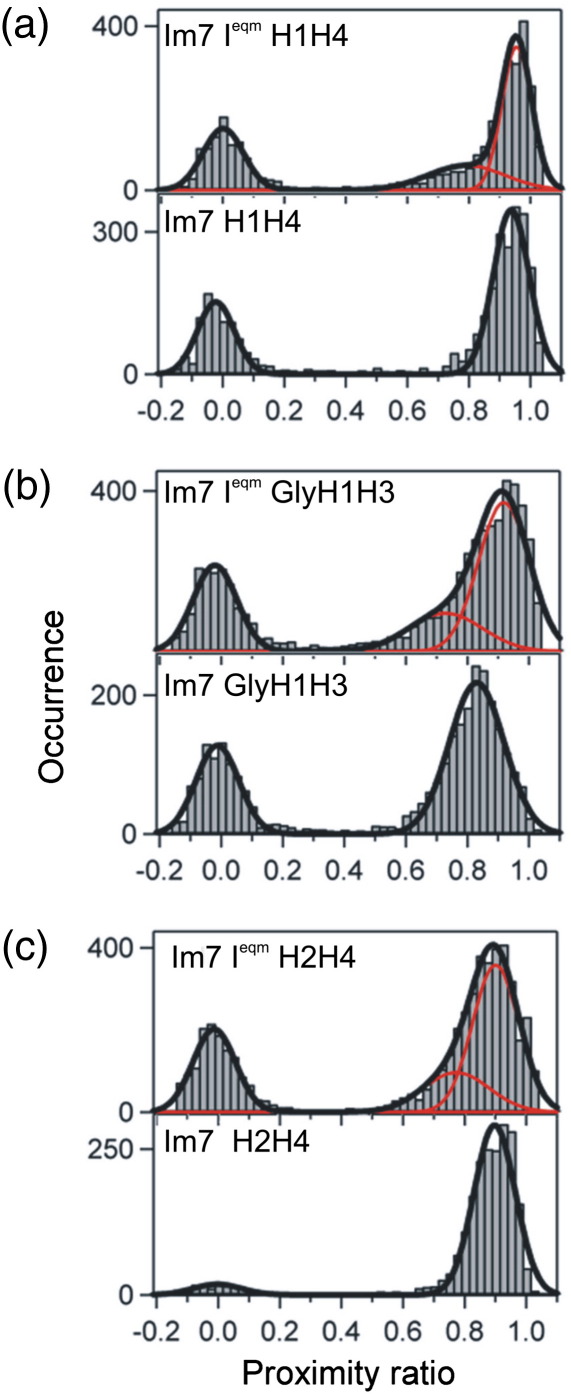
Proximity ratio histograms from smFRET measurements on Im7 and Im7 I^eqm^ species in 0 M urea with the fluorophores attached to (a) helices 1 and 4, (b) helices 1 and 3, and (c) helices 2 and 4 at pH 7, 10 °C with 0.4 M Na_2_SO_4_. Black lines show the fits obtained from summing one or two Gaussian distributions (red lines).

We first investigated the distribution of inter-dye distances for the three pairs of Im7 wild-type and I^eqm^ variants containing donor and acceptor dyes in different helical pairs using single-molecule diffusion experiments under conditions most commonly used for Im7 folding studies (0.4 M Na_2_SO_4_ at pH 7.0 and 10 °C). The FRET efficiency, herein referred as the proximity ratio or value (*P*, see Methods),^21,32^ was determined for each sample by measuring the number of detected donor and acceptor photons Id and Ia, respectively, from a single diffusing protein molecule in an integration time of 0.5 ms (see Methods). Histograms were then constructed of the *P* value, typically from 5000 such single molecules (Fig. 3). A peak centered on *P* = 0 of varying intensity is observed in all histograms and originates from proteins with a fluorescent donor but photobleached acceptor.^21^ This peak was ignored in subsequent analyses. Examination of the proximity ratio histograms for the native variants (bottom panels, Fig. 3a–c) shows a distribution at a high proximity ratio that fits well to a single Gaussian distribution, consistent with a single species being populated under these conditions. By contrast, the proximity ratio histograms for all the Im7 I^eqm^ variants show more complex behavior (top panels, Fig. 3a–c) and a second Gaussian is required to fit the data adequately. The peak at a higher proximity ratio in each case (*P* ≈ 0.90) likely reflects a folded conformation with short inter-helical separation populated by the intermediate. The peak at lower proximity ratio (*P* ≈ 0.75) reveals the presence of an ensemble of structures with a larger mean inter-dye separation reflecting the population of a more unfolded species.

Comparisons of the peak positions between the three helix pairs reveal further insights. For helices 1 and 4 (Fig. 3a), a highly populated species with *P* ≈ 0.93 for both Im7 H1H4 and Im7 I^eqm^ H1H4 is observed, indicative of efficient FRET and suggesting that these helices are closely packed in the trapped intermediate, consistent with previous MD simulations of this state.^10,14^ The mean proximity ratio of the most compact species for Im7 I^eqm^ H2H4 and for Im7 H2H4 (Fig. 3c) is also similar (*P* ≈ 0.90), implying that the distance between helices 2 and 4 in the intermediate ensemble, on average, is also native-like. For each of these helix pairs, the weight-averaged mean inter-helix distance for the intermediate ensemble derived from MD simulations^10,14^ (Fig. 1) is identical with the inter-helix distance in the native state (15.2 and 15.1 Å and 17.6 and 17.7 Å for Im7 H1H4 and Im7H2H4 variants, respectively, measured by the distance between the center of mass of each helix pair across the ensemble).^14^ By contrast, the positions of the folded peaks for Im7 GlyH1H3 and Im7 I^eqm^ GlyH1H3 (Fig. 3b) differ slightly, with mean proximity ratios *P* ≈ 0.82 and *P* ≈ 0.90, respectively. This suggests that helices 1 and 3 are, on average, in closer proximity in the intermediate state than in the native state and therefore in a nonnative relative orientation in at least the majority of molecules studied here. This is in contrast to the simulations that predict the weight-averaged separation to be greater in I^eqm^ and hence a smaller *P* value relative to the natively folded protein (18.0 and 14.7 Å, respectively).

In addition to revealing inter-dye distributions, analysis of proximity ratio histograms can provide insights into conformational dynamics.^21,27,32–34^ While the width of the proximity ratio distribution for a homogeneous and static species, such as the native state, is dominated by instrumental shot noise, additional broadening can indicate static conformational heterogeneity or inter-conversion between two or more distinct species on a time scale slower than, or similar to, the integration time used. The mean proximity ratios and width of the distributions of the trapped intermediate variants for the H1H4 and H2H4 pairs are indistinguishable from those of their wild-type analogues. This suggests little heterogeneity within the intermediate ensemble relative to its folded analogue.

### Trapped intermediates populate an unfolded ensemble in the absence of denaturant

**Fig. 4:**
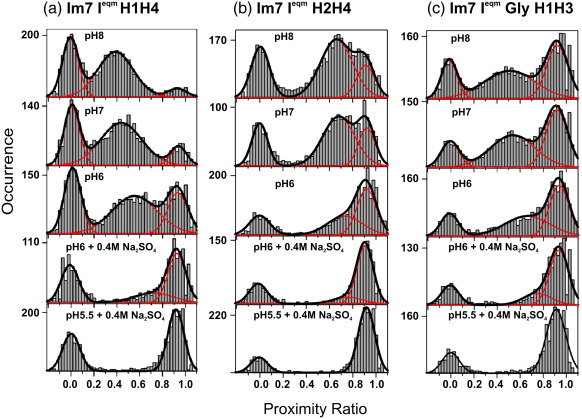
Proximity ratio histograms showing the effect of pH and Na_2_SO_4_ on the distribution of populated species in the I^eqm^ Im7 double-cysteine variants at 10 °C. As the pH is decreased and the concentration of Na_2_SO_4_ is increased, the unfolded state is depopulated and the intermediate state is populated for all variants studied. Black lines show the fits obtained from summing one or more Gaussian distributions (red lines).

Examination of the proximity ratio histograms for each of the I^eqm^ variants shows that an ensemble with a lower *P* value is co-populated with the folded state of each species (top panels, Fig. 3a–c), suggesting that an expanded or partially unfolded state is populated for these variants under native conditions. To elucidate the nature of this species, and to further study the folded members of the intermediate ensemble, we varied the solvent conditions from those known to favor a less-compact, more unfolded state (high-pH, low-sodium sulfate concentration) to those that have previously been shown to preferentially stabilize the intermediate state over the unfolded state (low-pH, high-sodium sulfate concentration).^35,36^ The proximity ratio histograms for the three Im7 I^eqm^ variants under these conditions are shown in Fig. 4. In all three cases, the relative population of the more highly unfolded species (lower *P* value) decreases with increasingly acidic conditions and/or in the presence of 0.4 M Na_2_SO_4_ while the more compact intermediate species concomitantly increases in a two-state transition. It may be expected that the ratio of the peak areas for the folded and unfolded species should reflect the relative stabilities of these variants, which are different as a consequence of the insertion of pairs of cysteine residues in different regions of the protein (Table 1). For example, in Fig. 4, at pH 6, the relative population of the unfolded to folded state is high for I^eqm^H1H4 while it is lower for both I^eqm^H2H4 and I^eqm^H1H3, which have similar stabilities. However, it should be noted that in our experiments, it is not molecules that are quantified but events in bins above a threshold. While these differences are actually accounted for in shot noise width analysis (see later), such effects complicate quantitative analysis of relative peak areas.

These data therefore support the assignment of the less-populated, lower-proximity-ratio species observed for the trapped intermediates to the unfolded state. It is noteworthy that the mean proximity ratio of the unfolded states of all the trapped intermediate variants shows a significant pH dependence, with apparent expansion as the pH is increased while the mean proximity ratios of the folded states of the trapped intermediate variants remain constant across the pH titration, obviating the pH dependence of the dyes as the origin of this effect. Note that both the donor and acceptor dye quantum yields have no significant pH dependence in the range 4–9.^37,38^ The isoelectric point of wild-type Im7 is ≈ pH 5. Therefore, as the pH is increased, it is plausible that the expansion in the unfolded state may be caused by electrostatic repulsion due to an increased negative charge. Importantly, at pH 7 and in the absence of Na_2_SO_4_, the unfolded state ensemble is highly populated for all I^eqm^ variants (Fig. 4, second row from the top). This provides the opportunity to study the unfolded ensemble of Im7 in weakly denaturing conditions, the initial starting species for the Im7 folding reaction.

### The unfolded Im7 ensemble becomes increasingly compact under mild denaturing conditions

**Fig. 5:**
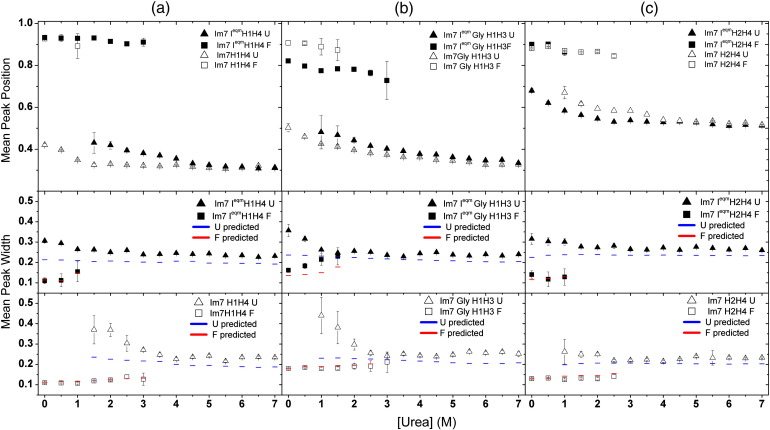
Mean proximity ratio and mean peak width for the folded and unfolded states for (a) Im7 H1H4 and Im7 I^eqm^H1H4, (b) Im7 GlyH1H3 and Im7 I^eqm^ GlyH1H3, and (c) Im7 H2H4 and Im7 I^eqm^H2H4 as a function of urea concentration at pH 7.0, 10 °C in the absence of Na_2_SO_4_. Triangles and squares represent the unfolded and folded states, respectively, and filled and open symbols correspond to the Im7 I^eqm^ and Im7 variants, respectively. The dotted blue and red lines represent the shot noise predictions for the unfolded and folded species, respectively. The error bars are ± 1 SD.

In order to explore the nature of the folded and unfolded species further, we performed a denaturant titration with the three Im7 and Im7 I^eqm^ helix pairs in the absence of kosmotrope. Shown in Fig. 5 are the mean proximity ratios and mean peak widths obtained from such titrations, using urea as the denaturant. The positions of the folded peaks (corrected for refractive index, see Methods) for all Im7 I^eqm^ and Im7 folded species (Fig. 5a, top panel, squares) change by less than *P* ≈ 0.06 as a function of urea concentration over the detectable range, suggesting little difference in compaction consistent with these ensembles representing stably structured states with a well-defined fold (note that given the large error, the data for Im7 I^eqm^ GlyH1H3 are not included in this analysis). Similar minor compaction with decreasing urea concentration has been previously observed for the homologous protein Im9 when labeled with dye at positions 23 and 81 (helix 1 and close to the C-terminus, respectively)^30^ as well as for the native states of other proteins.^39,40^ It is unlikely that these effects arise from restricted dye motion as anisotropy measurements suggest that each fluorophore has a high degree of conformational freedom. There is always a possibility that for a given set of labeling positions, the presence of urea changes the local environment of the dye in a way that causes some quenching due to the specific local sequence. In this regard, it has recently been shown that Im7 is a frustrated protein with a highly malleable core and this may lead to slight changes in conformation.^41^

In contrast with the behavior of the native and folded intermediate species, a substantial decrease in the inter-helical distance of the denatured state ensemble is observed as the denaturant concentration is decreased for all of the Im7 variants (Fig. 5a–c, top panels, triangles). In all cases, the effect is more pronounced than the changes observed for the folded ensembles, such that the *P* value changes by > 0.12 for all proteins between 0 and 6 M urea. Compaction of the unfolded state has been observed previously for other proteins at low concentrations of denaturant.^23,25,27,40,42–49^ Similar compaction of the denatured state at low denaturant concentration is observed with FRET donor–acceptor fluorophore pairs attached to the N- and C-termini of Im7 (data not shown), suggesting that compaction in the denatured state is isotropic with all regions of the protein showing similar behavior, rather than being confined to specific regions of the polypeptide chain as was observed with CspTm.^45^

As discussed above, the width of peaks in proximity ratio distributions contain information about dynamic conformational heterogeneity.^21,27,32–34^ The width of the distributions representing the native state or folded members of the intermediate ensemble (Fig. 5a–c, center and bottom panels, squares) is generally in excellent agreement with the expected shot noise limited width calculated as a function of denaturant concentration (Fig. 5a–c, center and bottom panels, broken lines, see Methods and Ref. 30). The peak width of the unfolded species for all variants (Fig. 5a–c, center and bottom panels, triangles) is independent of urea concentration under strongly denaturing conditions ([urea] > 3 M) but slightly broader than that predicted by shot noise (Fig. 5a–c, center and bottom panels, broken lines, see Methods). This broadness offset has also been observed for protein L,^34^ CspTm,^27^ and Im9.^30^ The unfolded state for all variants shows significant additional broadening in the transition region, where both the folded state and unfolded state are populated (Fig. 5a–c, center and bottom panels, triangles). These findings suggest that the unfolded state ensembles of all six variants become not only more heterogeneous but also, on average, more compact, in weakly denaturing conditions, consistent with slowed fluctuations relative to the 0.5-ms measurement time between isotropically collapsed ensembles of conformationally heterogeneous states that occur under conditions favoring folding.

### The properties of the native, intermediate, and unfolded ensembles in the presence of sodium sulfate

**Fig. 6:**
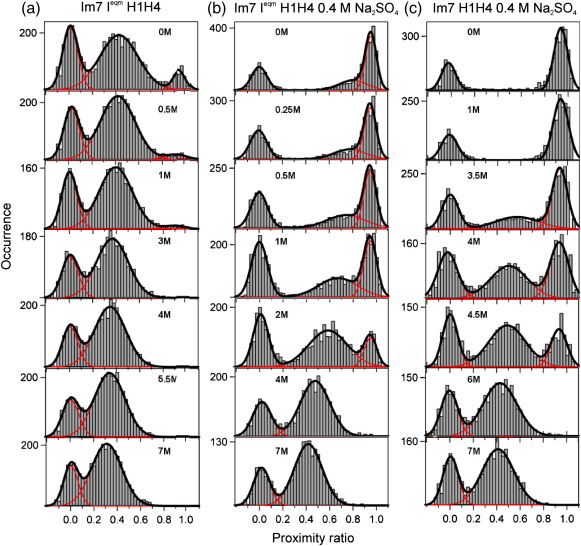
Selected proximity ratio histograms of the equilibrium urea denaturation of (a) Im7 I^eqm^H1H4 at pH 7 in the absence of Na_2_S0_4_ and (b) Im7 I^eqm^H1H4 and (c) Im7H1H4 at pH 7 and 0.4 M Na_2_S0_4,_ 10 °C monitored by smFRET. Black lines show the fits obtained from summing one or more Gaussian distributions (red lines).

**Fig. 7:**
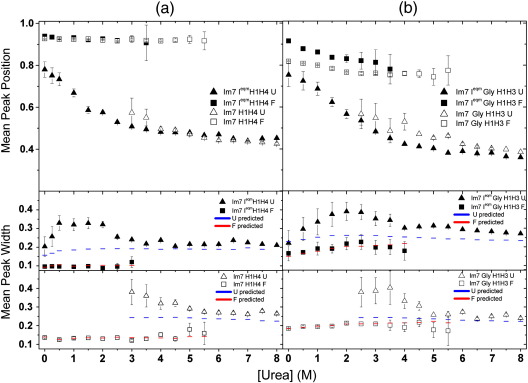
Mean proximity ratio and mean peak width for the folded and unfolded species of (a) Im7 H1H4 and Im7 I^eqm^H1H4 and (b) Im7 GlyH1H3 and Im7 I^eqm^ GlyH1H3 as a function of urea concentration at pH 7.0 and 0.4 M Na_2_SO_4_, 10 °C. Triangles and squares represent the unfolded and folded species, respectively, and filled and open symbols correspond to the Im7 I^eqm^ and Im7 variants, respectively. The dotted blue and red lines represent the shot noise predictions for the unfolded and folded species, respectively. The error bars are ± 1 SD.

Further studies of the properties of the folded and unfolded states of Im7 H1H4 and Im7 GlyH1H3 and their analogous trapped intermediate constructs were performed in the presence of a kosmotrope (0.4 M Na_2_SO_4_, sample raw data are shown in Fig. 6 and summarized in Fig. 7). Studies on Im7 I^eqm^ H2H4 and Im7 H2H4 were not included, as the presence of 0.4 M Na_2_SO_4_ results in poor separation of the distributions corresponding to the folded and unfolded species in the transition region, ruling out their quantitative analysis (data not shown). The stabilizing effect of the kosmotrope results in the folded intermediate species of Im7 I^eqm^ H1H4 and Im7 I^eqm^ GlyH1H3 being populated up to 3 M urea and the native states of Im7 H1H4 and Im7 GlyH1H3 up to 5.5 M urea (Fig. 7a, squares). This allows the effect of denaturant on the inter-helical distance (*P* value) and distribution width of these species to be quantified over a wider range of urea concentration than was possible hitherto. Similar to the denaturant titration in the absence of kosmotrope, no significant compaction is observed for the folded Im7 H1H4 and the folded Im7 I^eqm^ H1H4 conformations as denaturant concentration is decreased (Fig. 7a, top panel, squares). For the Im7 GlyH1H3 variants, contrasting behavior is seen: compaction occurs towards low denaturant concentrations, which was unclear in the absence of kosmotrope (Fig. 5b, top panel, squares, and Fig. 7b, top panels, squares) with a change in *P* value by up to 0.13 from 0 to 3.5 M urea. The cause of this apparent compaction is unclear and may be a consequence of the inserted glycine sequence and will require structural data with higher resolution to resolve. While differences are observed in inter-helical distance, the widths of both the H1H4 and GlyH1H3 native and intermediate folded ensembles remains shot noise limited across the entire denaturant range studied (Fig. 7a and b, middle and bottom panels, squares). This suggests that despite the significant compaction seen in the GlyH1H3 native and folded intermediate, the ensemble of interactions between helices 1 and 3 and 1 and 4 have native-like dynamics and homogeneity, irrespective of the concentration of denaturant.

The unfolded state ensembles of the proteins Im7 H1H4 and Im7 Gly H1H3 and their trapped intermediate analogues in the presence of 0.4 M Na_2_SO_4_ show a dramatic dependence of their inter-helical separation with denaturant concentration (Fig. 7a and b, top panels, triangles). Strikingly, in 0 M urea, the unfolded state ensemble becomes so compact_,_ (*P* ≈ 0.8) that its *P* ratio is close to that of the native ensemble. Correlated with this, a dramatic and highly significant change in the width of the unfolded state of the trapped intermediate is observed in mildly denaturing conditions (Fig. 7a and b, center panels, triangles), which is not observed for the folded state observed in the same experiment, obviating instrumental or processing artifacts as the cause.^34^ For both Im7 I^eqm^H1H4 and Im7 I^eqm^GlyH1H3 at denaturant concentrations between 1 and 3 M, the distribution width increases as the inter-dye separation decreases. The distribution width of the unfolded peak then decreases again as the concentration of urea is decreased below 1 M, possibly reflecting a decreased rate of conformational exchange among unfolded but compact conformations.

## Discussion

The results of this single-molecule study uncover new insights into the properties of the different species populated during the folding of Im7. The peak positions and distribution widths for the natively folded Im7 H1H4, Im7 H1H3, and Im7 H2H4 variants are narrow and at high *P* values as expected based on the known structure of Im7.^13^ Comparison of these data with those for their trapped intermediate analogues allows differences in the structure and dynamics of each state to be identified. Analysis of the proximity ratios between helix pairs suggests that the separations between helices 1–4 and 2–4 are indistinguishable between the native and folded intermediate states, at least for the positions measured. Interestingly, the proximity ratio of folded Im7 I^eqm^ GlyH1H3 is slightly higher than that of Im7 GlyH1H3 (*P* ≈ 0.90 and 0.82, respectively, Figs. 3b and 7b, top panel), indicating a shorter inter-residue distance, on average, for residues 17 and 56 in the intermediate ensemble, possibly consistent with this region being unstructured and occupying a highly nonnative location within the intermediate ensemble.^10,15^ φ value analysis for the intermediate state of Im7 revealed nonnative interactions between side chains in helices 2 and 4, specifically involving residues towards the C-terminal end of helix 2, including residues F41 and V42.^16^ Presumably then, the changes in side-chain packing needed to reach the native state involve subtle reorganization rather than large-scale inter-helix movements that would have been detected in the smFRET studies described here. These data accord with the analysis of the trapped intermediate by NMR, which found native-like secondary structure in helices 1 and 4, a partial formation of helix 2, and the absence of a structured helix 3.^20^

The data also allow benchmarking of the ensemble of intermediate states calculated using MD simulations.^10,14^ For Im7 H1H4 variants, there is excellent agreement; both techniques suggest that the arrangement of this helix pair is similar in both the native and intermediate states and there is little structural heterogeneity in this equilibrium ensemble. In this study, a similar result was observed for the Im7 H2H4 variants. This is in contrast to MD simulations of the intermediate ensemble that predicts a broader distribution of distances for the helix 2–4 pair than in the native state.^14^ Interestingly, however, comparison of the weight-averaged helix–helix distance for helices 2–4 calculated from the simulations demonstrates that these distances are identical for the native and folded intermediate state (17.7 and 17.6 Å, respectively). If the intermediate ensemble predicted by simulation is accurate, then this suggests that the conformational heterogeneity observed between helices 2–4 results from conformational exchange on a timescale faster than the timescale of the single-molecule experiment presented here.

The smFRET experiments also enabled the properties of the unfolded, intermediate, and native states to be determined as a function of the concentration of urea. Generally, the native and folded intermediate species were found to be only marginally compacted, as judged by the proximity ratio, as the denaturant concentration decreased, and were unaffected by the presence of 0.4 M Na_2_SO_4_ in agreement with previous studies using equilibrium denaturation.^35,36^ The peak width for the native and folded intermediate states was also generally found to be insensitive to denaturant concentration with the exception of Im7 I^eqm^ GlyH1H3 in the absence of 0.4 M Na_2_SO_4_. Introduction of cysteine pairs into Im7 resulted in destabilization of both the native state and the trapped intermediate state (I^eqm^ variants) by ≤ 4.5 and ≤ 3.3 kJ mol^− 1^, respectively. This proved to be advantageous, allowing the characterization of the unfolded state at very low concentrations of denaturant. This allowed observation of the equilibrium collapse (coil–globule transition) that is usually masked by the folding transition. Our data reveal that substantial compaction of the unfolded state occurs, as predicted by Alonso and Dill^50^ and observed previously for several other proteins using both single-molecule fluorescence^25,27,42,44,45,51^ and other techniques.^51–54^ Such data have been modeled as a continuum of substates^25^ or by using an analytical polymer model.^51^ In the latter approach, smFRET data reporting on the coil–globule transition were used to calculate the end-to-end distance probability distribution for several proteins as a function of interaction energy (or denaturant concentration). These data demonstrated a continuous contraction and narrowing of the distribution as denaturant concentration decreases. Conversion of these data to radii of gyration allowed the expansion of the denatured state relative to the native state to be extrapolated to the absence of denaturant where the unfolded state was found to be only 30% larger than that of the folded state,^51^ akin to the significant collapse observed in this study in the absence of urea. Furthermore, the free energy of denatured state collapse has a linear dependence on denaturant concentration with a similar gradient to that for the folding reaction, suggesting that the effect of denaturant is mediated through the collapse transition of the denatured state.^55^

At low concentrations of denaturant, the proximity distribution width for the unfolded states of each variant is also dependent on the denaturant concentration (Fig. 5), possibly due to slower exchange between iso-energetic conformations in the unfolded energy basin at low chaotrope concentrations.

In the presence of 0.4 M Na_2_SO_4_, the unfolded states of Im7 I^eqm^ H1H4 and Im7 I^eqm^ Gly H1H3 are populated even in the absence of denaturant, and a striking new observation is possible (Fig. 7a and b, middle panel, triangles): After an initial increase in the width of the unfolded ensemble as described above, in mild denaturing conditions, the width narrows again as the urea concentration approaches zero (Fig. 7), reflecting the preferential population of a more homogeneous or less dynamic unfolded conformation. It is possible that both the compact unfolded state in 0.4 M Na_2_SO_4_ and the more expanded unfolded species in the absence of Na_2_SO_4_ belong to the same unfolded ensemble (i.e., they are not distinct thermodynamic states). In such a scenario, the addition of Na_2_SO_4_ would preferentially stabilize the more compact unfolded species, resulting in conformations with higher *P* ratios dominating the unfolded ensemble. The precise nature of the interactions that stabilize these compact states is still debated and could involve either specific or nonspecific hydrophobic interactions.^51,53,55^ Such highly compact, unfolded states could be favorable for efficient folding, limiting the conformational search to the native structure. Further studies by NMR will be needed to determine the properties of this state in atomistic detail, akin to the analyses of other nonnative species of Im7 and other proteins.^18,20,56–59^ Severely destabilized variants of Im7, where the unfolded state is populated in the presence of kosmotrope and in the absence of denaturant, may provide an ideal starting point for such studies.

## Methods

### Chemicals and reagents

Alexa Fluor 488 and 594 C-5 maleimide were purchased from Invitrogen (UK). Fluka brand reagents (Sigma-Aldrich, UK) were used for all single-molecule measurements. Urea was recrystallized in analytical-grade ethanol prior to use.

### Protein engineering

The desired mutations were introduced into hexahistidine-tagged Im7^16^ using QuikChange Site-Directed Mutagenesis Kit (Stratagene, UK). Im7 double-cysteine variants are named using the wild-type residue number (ignoring the His-tag) and the residue to which it has been mutated (e.g., K70C is lysine 70 mutated to cysteine). The proteins were purified to homogeneity as previously described^36^ and their identity was verified using mass spectrometry.

### Characterization of the unlabeled protein

Fluorescence emission spectra were recorded in a Photon Technologies International Quantamaster C-61 spectrofluorimeter at 10 °C using protein at a concentration of ≈ 5 μM in 50 mM sodium phosphate buffer, pH 7.0, 0.4 M Na_2_SO_4_, 1 mM ethylenediaminetetraacetic acid, and 4 mM DTT and in the same buffer containing 8 M urea. The fluorescence of tryptophan and tyrosine residues was excited at 280 nm, and emission spectra were collected with a scan rate of 1 nm/s between 300 and 450 nm. Buffer blanks were subtracted and the spectra were normalized to the emission maximum of the unfolded state in 8 M urea.

### Ensemble equilibrium denaturation of unlabeled protein

The stability of the Im7 double-cysteine variants was determined by equilibrium denaturation using urea titration. The samples were made from stock solutions of buffers containing 50 mM sodium phosphate buffer, pH 7.0, 0.4 M Na_2_SO_4_, 1 mM ethylenediaminetetraacetic acid, and 4 mM DTT in the absence or presence of 9 M urea. The solutions were mixed in appropriate proportions to give final urea concentrations ranging from 0 to 8 M (in 0.2-M increments) and a final protein concentration of ∼ 5 μM. Time-based fluorescence measurements were performed at 10 °C using a Photon Technologies International Quantamaster C-61 spectrofluorimeter. The samples were excited at 280 nm, and the emitted light at 360 nm was measured over a 60-s period. After signal averaging, we plotted the intensity as a function of urea concentration, and the data were fitted to a two-state transition as described previously.^5^ To compare the denaturation profiles of the variants, we converted the raw data to fraction population of native molecules.^60^

### Labeling with fluorophores

Each double-cysteine variant at a concentration of 3 mg/ml was first labeled with a 0.65 molar ratio of Alexa Fluor 594 C-5 maleimide in 50 mM sodium phosphate and 10% dimethyl sulfoxide, pH 7.3 (labeling buffer), for 45 min at room temperature. Singly labeled protein was separated from unlabeled and doubly labeled protein by anion-exchange chromatography using a prepacked 6 ml Resource Q anion-exchange column on an ÄKTA explorer system equilibrated with 50 mM sodium phosphate pH 7.3 (buffer A). Proteins were eluted with 0–50% gradient over 14 column volumes using buffer A with 1 M NaCl. Singly labeled protein at a concentration of 1 mg/ml in 6 M urea was then labeled with a 2-fold molar excess (over the thiol concentration) of Alexa Fluor 488 C-5 maleimide in labeling buffer for 2 h at room temperature. Double-labeled protein was separated from singly labeled protein by anion-exchange chromatography as described above. This procedure allows the preparation of highly pure proteins, each labeled with a single donor and acceptor pair, but results in an A/B, B/A mix. Remaining traces of unreacted dye were removed by gel filtration using a Superdex Peptide HR 10/30 column in 50 mM sodium phosphate buffer, pH 7.0. At each labeling step, the identity of the product was confirmed by electrospray ionization mass spectrometry.

### Fluorescence anisotropy

Steady-state anisotropy data were measured using a HORIBA Jobin Yvon Fluorolog spectrophotometer with double-labeled protein solution at a concentration of 100 nM at 10 °C in 50 mM sodium phosphate buffer, pH 7.0 in the presence or absence of 8 M urea. Each sample was measured with excitation at both 488 and 594 nm, 60 times over the course of 1 min. This was repeated three times, and the mean anisotropy value was calculated.

### Single-molecule FRET

Single-molecule experiments were performed using a custom-built confocal microscope described in Refs. 21, 30, and 61. Solutions contained 0–8 M urea (in increments of 0.5 M) in 50 mM sodium phosphate buffer, pH 7.0, 0.01% (w/v) Tween 20 with 0.4 M Na_2_SO_4_, unless otherwise stated. In addition, 1.5 mM l-carnosine, 2 mM mercaptoethanol, and 10 mM (1,4-diazabicyclo[2,2,2] octane were included as oxygen scavengers and to suppress blinking, to minimize the magnitude of the zero peak. Singlet oxygen and dark states (possible triplet-state population) are thought to be the main cause of premature photobleaching of the acceptor dye. Inclusion of these compounds at the stated concentrations has no effect on the thermodynamic parameters (*M*_un_ and Δ*G*_un_°) obtained for this protein.

The protein concentration of each sample was ≈ 50 pM, and all measurements were performed at 10 °C. Data were collected by observing the transient bursts of fluorescence produced by diffusion of single molecules into and out of the confocal detection volume, using an integration time of 0.5 ms. Ratiometric analysis of single-molecule data was performed as described in Ref. 30 using custom algorithms written in the data analysis software package Igor Pro Version 5.06a (Wavemetrics Inc., USA). The proximity ratio, *P*, was calculated using;

(1) $$P=\frac{\left(I_{\text{A}}-\langle B_{\text{A}}\rangle -\langle \varphi I_{\text{D}}\rangle \right)}{\left(I_{\text{A}}-\langle B_{\text{A}}\rangle -\langle \varphi I_{\text{D}}\rangle \right)+\left(I_{\text{D}}-\langle B_{\text{D}}\rangle \right)}$$

where *I*_A_ and *I*_D_ are the acceptor and donor signals in each 0.5-ms interval, respectively, 〈*B*_A_〉 and 〈*B*_D_〉 are the mean background signals for the acceptor and donor channels, respectively, and φ is the mean cross-talk (leakage) of fluorescence from the donor into the acceptor channel, determined to be ≈ 10% in a separate experiment using a concentrated donor-only sample (data not shown). Note that the FRET efficiency *E* is calculated from the ratio of the detected acceptor signal to the total signal (donor + acceptor) in the same integration time:

(2) $$E_{\text{FRET}}=\frac{I_{\text{A}}}{\gamma I_{\text{D}}+I_{\text{A}}}$$

where *I*_A_ and *I*_D_ are the uncorrected number of donor and acceptor photon counts per counting interval. In this study, the ratio γ was assumed to be equal to 1. In this limit, a proximity ratio is the FRET efficiency and can be converted, if required to a distance (*R*_0_ = 54 Å for the dye pair used in this study).^27^

A SUM threshold criterion was then applied to the data in order to select only integration times that contained valid single-molecule events and to reject the background;^21,32^

(3) $$\left(I_{\text{A}}-\langle B_{A}\rangle -\langle \varphi I_{\text{D}}\rangle \right)+\left(I_{\text{D}}-\langle B_{\text{D}}\rangle \right)\geq T,$$

where *T* is the particular threshold used. Histograms of the accepted proximity ratios were then constructed and fitted with the sum of two or three Gaussians with the following formularization:

(4) $$\text{Occurrence}(p)=\sum_{n=1}^{n=3}\frac{a_{n}}{w_{n}\sqrt{\pi /2}}\exp\left(\frac{-2{\left(p-p_{0,n}\right)}^{2}}{w_{n}^{2}}\right)$$

where *a*_*n*_ is the area under the curve, from the baseline (fixed = 0) of curve *n*. *w*_*n*_ = 2σ_*n*_, where σ_*n*_ is the standard deviation of curve *n*. *p*_0,*n*_ is the mean proximity ratio (peak top for a Gaussian) of curve *n*. Fits were otherwise unconstrained. The mean proximity ratio values obtained from the fits were corrected for changes in the average refractive index of the solution (due to different urea concentrations) following Refs. 27 and 30.

### Peak width analysis

Peak width analysis was performed as described in Ref. 30 using a normalized formularization of the expected shot noise distribution with proximity ratio^32^ and using algorithms written in Igor 5. The predicted shot noise limited width of a given species is then:

(5) $$2\sigma (m,S)=2N\frac{\sqrt{m(1-m)}}{\sqrt{S+1}}$$

where σ is the standard deviation, *m* is the mean proximity ratio of the species, *S* is the mean total signal of the identified single-molecule events in that species, obtained by analysis of the actual single-molecule bursts contributing to that species, and *N* is a normalization factor. Using *N* = 1, the width of the species of interest, in this case the folded peak, is underestimated, as previously described.^30^ Assuming that the native state of the labeled Im7 variants can be described as a homogeneous, static species, their width therefore represents the shot noise limit for our particular set of experimental conditions. Therefore, for each of the native peaks of the three variants, Im7 H1H4, Im7 H2H4, and Im7 Gly H1H3 in 0 M urea, a normalization pre-factor was generated. The pre-factor was then used to determine the expected shot noise contribution at all urea concentrations for both the native and denatured ensembles, of each Im7 helix pair, using Eq. (5).^30^ In order to investigate the widths of the folded and unfolded peaks of the labeled trapped intermediate proteins as a function of urea concentration, we used the pre-normalization factor generated using the wild-type variant with fluorophores in identical locations. The reasoning behind this approach is that the intermediate may be heterogeneous and dynamic; therefore, the width of this species cannot be assumed to be due to shot noise alone.

## Figures and Tables

**Table 1:** Biophysical characterization of the Im7 variants used in this study (at pH 7, 10 °C)

Variant	Δ*G*_un_° (kJ mol^− 1^)^a^	*M*_un_ (kJ mol^− 1^ M^− 1^)^a^	Anisotropy			
			Excitation^b^		Emission^c^	
			0 M urea	8 M urea	0 M urea	8 M urea
Im7	24.6 ± 1.3	4.9 ± 0.7	ND	ND	ND	ND
Im7 GlyH1H3	22.4 ± 0.8	5.6 ± 0.1^d^	0.11 ± 0.07	0.11 ± 0.03	0.17 ± 0.05	0.13 ± 0.08
Im7 H2H4	20.1 ± 0.8	4.7 ± 0.1	0.15 ± 0.07	0.12 ± 0.06	0.14 ± 0.02	0.12 ± 0.05
Im7 H1H4	20.8 ± 0.6	4.9 ± 0.1	0.10 ± 0.06	0.11 ± 0.05	0.14 ± 0.03	0.12 ± 0.04
Im7 I^eqm^	10.1 ± 0.3^e^	3.4 ± 0.2^e^	ND	ND	ND	ND
Im7 I^eqm^ GlyH1H3	7.9 ± 0.9	4.5 ± 0.3^d^	0.12 ± 0.08	0.11 ± 0.05	0.17 ± 0.06	0.10 ± 0.05
Im7 I^eqm^ H2H4	7.8 ± 1.2	3.5 ± 0.3	0.18 ± 0.07	0.12 ± 0.03	0.17 ± 0.04	0.12 ± 0.03
Im7 I^eqm^ H1H4	6.8 ± 1.1	3.8 ± 0.2	0.19 ± 0.07	0.11 ± 0.06	0.17 ± 0.02	0.11 ± 0.03

The thermodynamic parameters (Δ*G*_un_° and *M*_un_) were calculated from chemical denaturant equilibrium unfolding experiments. Errors are those from fits of the data to a two-state equilibrium model.

a Measured in the presence of 0.4 M Na_2_SO_4_.

b Anisotropy of donor fluorophore measured at 488 nm.

c Anisotropy of acceptor fluorophore measured at 594 nm.

d *M* values for the two GlyH1H3 variants are significantly larger than their analogues without glycine insertions, consistent with the introduction of 15 glycine residues.

e Taken from Ref. 19.
